# Interventions to improve neurocognitive late-effects in pediatric and adolescent CNS tumor patients and survivors - a systematic review

**DOI:** 10.3389/fonc.2023.1150166

**Published:** 2023-05-02

**Authors:** Rahel Kasteler, Philipp Fuchs, Maria Otth, Katrin Scheinemann

**Affiliations:** ^1^ Division of Pediatric Hematology/Oncology, Department of Pediatrics, Kantonsspital Aarau, Aarau, Switzerland; ^2^ Department of Oncology, Hematology, Immunology, Stem Cell Transplantation and Somatic Gene Therapy, University Children’s Hospital Zurich, Zurich, Switzerland; ^3^ Division of Pediatric Hematology/Oncology, Children’s Hospital of Eastern Switzerland, St. Gallen, Switzerland; ^4^ Department of Health Sciences and Medicine, University of Lucerne, Lucerne, Switzerland; ^5^ Division of Pediatric Hematology/Oncology, McMaster Children’s Hospital and McMaster University, Hamilton, ON, Canada

**Keywords:** late-effects, pediatric brain tumors, intervention, neurocognition, children, adolescent and young adult

## Abstract

**Introduction:**

Survival of children and adolescents diagnosed with central nervous system (CNS) tumors massively improved over the last decades due to better diagnostics, treatment, and supportive care. However, morbidity is still the highest of all cancer entities in this age group with neurocognitive late-effects being one of the most severe.

**Aim:**

With this systematic review, we aim to summarize interventions designed to prevent or improve neurocognitive late-effects in CNS tumor patients.

**Method:**

We searched PubMed on August 16^th^ 2022 and included publications studying interventions for neurocognitive late-effects in pediatric and adolescent patients and survivors diagnosed with a CNS tumor. We included any form of neurocognitive intervention during treatment or following treatment completion. We considered all types of studies except for expert opinions and case reports.

**Results:**

The literature search resulted in 735 publications. We included 43 publications in the full text screening and 14 met our inclusion criteria. Of those, two assessed the impact of pharmacological interventions, three of exercise interventions, five of online cognitive training, and four assessed behavioral interventions. Different neuropsychological test batteries and imaging were used to measure the impact of the respective interventions. Most studies showed a positive impact of the interventions in single to several of the subtests used.

**Conclusion:**

We found several intervention studies indicating improvement of neurocognitive problems in children and adolescent CNS tumor survivors. In this population exercise interventions or online cognitive training might mitigate or improve neurocognitive late-effects.

## 1 Introduction

Improvement in diagnostics, treatment, and supportive care of pediatric and adolescent central nervous system (CNS) tumor patients led to an increase in survival over the last decades (1, 2). Morbidity in pediatric, adolescent and adult CNS tumor survivors is still very high compared to other cancer entities (3–5). This is owed to the location of the cancer itself and to the severity of the treatment modalities, including a combination of chemotherapy, neurosurgery and radiotherapy to the brain (6). Neurocognitive late-effects are commonly reported to be of the most severe ones and can impact long-term development and impair life goals such as education, employment or independence (3, 7). Thus, interventions to mitigate neurocognitive late-effects in CNS tumor survivors are urgently needed. With this systematic review, we aim to summarize interventions designed to improve neurocognitive late-effects in pediatric or adolescent CNS tumor patients or survivors.

## 2 Methods

We performed this review according to the PRISMA guidelines for reporting systematic reviews and meta-analyses (8).

### 2.1 Literature search

We conducted a systematic literature search in PubMed on August 16^th^ 2022. We did not restrict the search for date of publication or language. We built the search strategy around six concepts according to the PICO framework (**Supplementary Table 1**) (9). We identified MeSH terms and free text words for each concept, which we finally combined. For the population of interest, we included the concepts of “central nervous system tumors” (including specific types of CNS tumors but also broad terms), “children and adolescents”, “survivors” and “patients”. For the outcome, we chose terms related to “neurocognition” and “interventions”. For each review article identified with our search, we screened the reference list for relevant original articles.

### 2.2 Inclusion and exclusion criteria

To be included in this review, the studies had to report on cancer patients or survivors who were diagnosed with a CNS tumor during childhood or adolescence, and who received any form of neurocognitive intervention during treatment or following treatment completion. For a study cohort to be considered “children or adolescents”, at least 75% of participants had to be aged less than 18 years at the time of the cancer diagnosis. We excluded studies that did not fulfil the inclusion criteria, case reports, case series (n ≤ 14), commentaries, editorial letters, poster abstracts and review articles.

### 2.3 Outcomes

The primary outcome of this review was to describe neurocognitive interventions applied in children and adolescents diagnosed with CNS tumors. The impact of the neurocognitive interventions on neurocognition was the secondary outcome.

### 2.4 Data extraction and quality assessment

Two reviewers (MO and KS) screened all titles and abstracts separately and excluded those not fulfilling the inclusion criteria. Any disagreements were discussed and resolved by a third reviewer (RK). Two other reviewers (RK and PF) independently checked all retrieved full texts for adherence. Disagreement was resolved by a third reviewer (MO). Data from the eligible studies were extracted to a standard sheet including the first author, year of publication, patients’ age at diagnosis, sample size, study design, type of neurocognitive intervention, and the impact of the intervention on neurocognition, if reported. We summarized the neurocognitive interventions thematically. We assessed the quality, relevance and reliability of each included study by using the appropriate critical appraisal tool from the Joanna Briggs Institute (10), including the checklists for randomized controlled trials and quasi-experimental studies (**Supplementary Table 2**). Since these tools do not use any categorization, we made a classification with three categories. If all criteria of the respective checklist were fulfilled, we assigned the study to “Quality 1”. If a controlled trial was not truly randomized or did not use a crossover design to overcome differences in study group characteristics, we assigned the study to “Quality 2”. If a quasi-experimental study had no control group, the study was assigned to “Quality 2”. If an additional point from the checklist for both types of studies was insufficiently covered, we assigned the study to “Quality 3”.

## 3 Results

**Figure 1:**
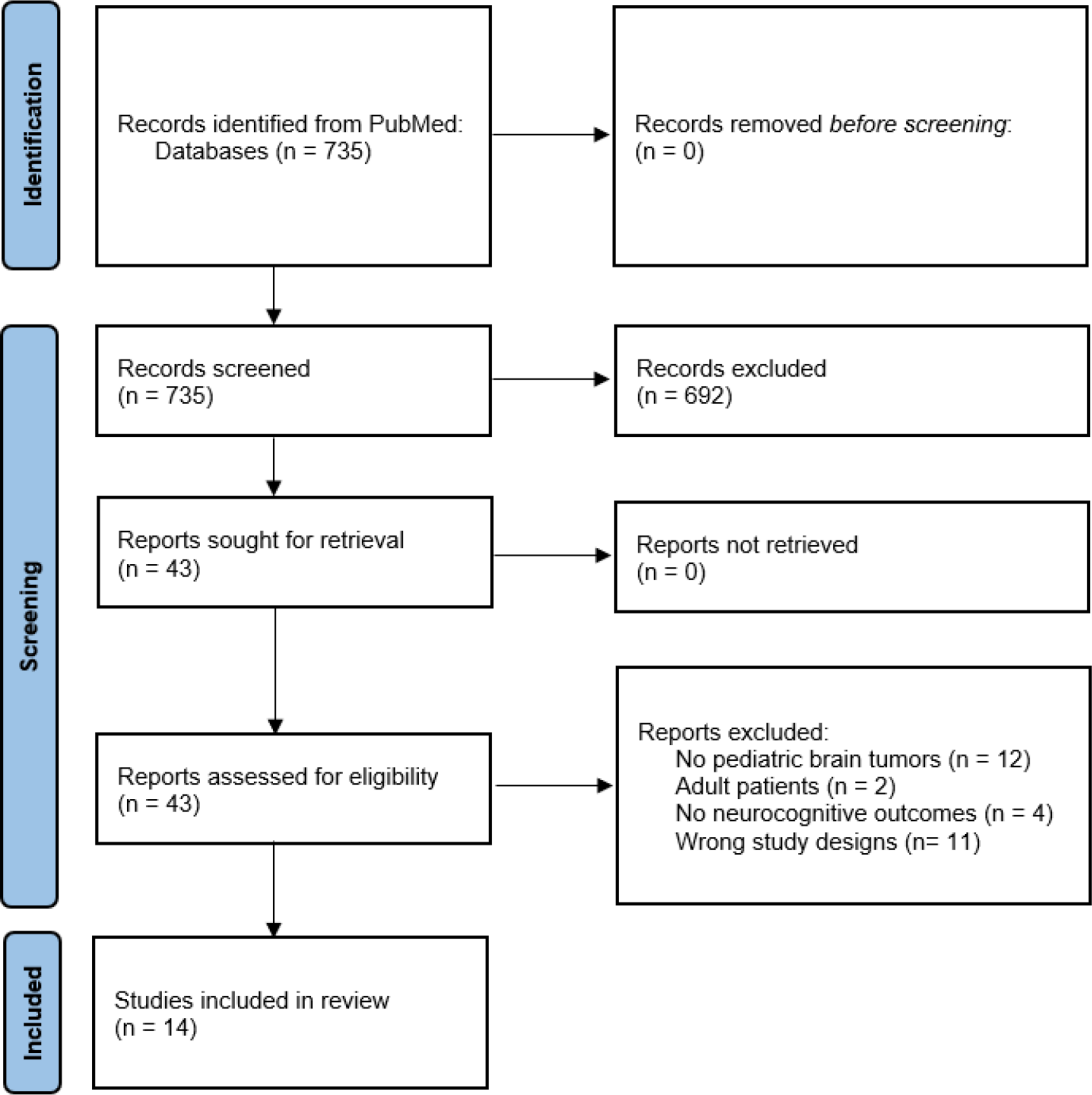
PRISMA 2020 flow diagram for systematic review.

Of 735 potentially relevant publications resulting from our literature search, we excluded 692 publications after title and abstract screening and included 43 for full text screening. Fourteen publications met our inclusion criteria (**Figure 1** and **Table 1**), 12 did not report on pediatric brain tumors, two were on adult patients or mixed populations only, four reported on other outcomes, and 11 were the wrong study designs (e.g. reviews). Regarding the patient population, most studies did not reflect the typical distribution of pediatric and adolescent CNS tumors but described selected groups of patients or survivors. Astrocytoma, the most frequent cancer type often accounted for a small proportion only. Medulloblastoma was the most frequent diagnosis reported followed by ependymoma. Among the 14 included studies, only two were double-blind, placebo controlled randomized controlled trials (RCTs) (11, 12), one of them with crossover design (11). Three studies were not blinded RCTs (13–15), one of them had a crossover design (14). Four studies had a quasi-randomized controlled crossover design (16–19), and two studies were controlled intervention trials (20, 21). Three studies were single-arm intervention trials without a control-group (22–24). In the quality assessment only one study met all quality criteria and was thus graded Quality 1 (12). Three studies were categorized as quasi-experimental studies due to lack of control groups (22–24) and were rated Quality 2 as they met most quality criteria. The remaining studies were rated Quality 3 because additional quality criteria were not met or not reported (**Table 1**). Even though in most included studies neuropsychological tests were performed to assess the outcomes, the test batteries and subtests used were different. Memory was assessed by Automated Working Memory Assessment (AWMA) (22), Cambridge Neuropsychological Test Automated Battery (CANTAB) (17–19), Children’s Auditory Verbal Learning Test-2 (CAVLT-2) (11), List Sorting Working Memory (LSWM) (11, 15), Picture Sequence Memory Test (PSM) from the NIH toolbox (11, 15), Rey Auditory Verbal Learning Test (RAVLT) (11, 14), Wechsler Intelligence Scale for Children (WISC-III/IV) (12, 14, 20), or Wide Range Assessment of Memory and Learning (WRAML-2) (20, 23). Processing speed and attention was assessed mainly by the CANTAB test battery (11, 18, 19). Other tests used to assess processing speed were WISC IV (14, 23), mean reaction time on correct answers on Attention Network Tasks (ANT) (12), or Pattern Comparison Processing Speed Test from the NIH toolbox (15). Other tests used to assess attention were Conners Continuous Performance Test (CPT II) (14, 23), Map Mission (14), visual scanning from Dellis-Kaplan Executive Function Test (D-KEFS) (14), or ANT (12). Executive functioning was assessed by Behavior Rating Inventory of Executive Function (BRIEF) (24), D-KEFS (14), or Flanker Inhibitory Control and Attention Test from the NIH toolbox (15). Two studies used imaging findings to assess the impact of the intervention (18, 19).

**Table 1:** Study characteristics of included publications.

Author, Year of publication, Study identifier	N	Age at diagnosis [years] (mean; SD (range))	Age at intervention [years] (mean; SD (range))	Diagnoses (n)	Study design; Quality
Ayoub et al., 2020, NCT02040376	23	G1: 7.3; 3.3 (2–13) G2: 6.4; 3.6 (1–13)	G1: 14.9; 3.5 (8-20) G2: 14.1; 3.1 (10-20)	Medulloblastoma (12) Germ cell (3) Ependymoma (2) Others (6)	Pilot RCT, double-blind, crossover; Quality 2
Castellino et al., 2012, NCT00452868	11	Age at cranial RT: 5.6 (4.2-13.2)	RT to study enrolment: 4.7 (1.9–11.9) (median (range))	Medulloblastoma (7) Others (4)	Single arm intervention trial; Quality 2
Cox et al., 2020, NCT01944761	20	G1: 6.6; 1.1 (9.7-16.9) G2: 5.8; 2.8; (1.9-9.3)	G1: 12.5; 2.9 (9.7-16.9) G2: 11.1; 3.2 (7.4-16.9)	Medulloblastoma (14) Ependymoma (5) (incl. anaplastic = 2) Germ Cell (2)	CT, crossover; Quality 3
Riggs et al., 2017, NCT01944761	28	G1: 6.3; 1.6 (2.9-8.1) G2: 5.6; 2.6 (1.9-9.3)	G1: 12; 3 (8.1-16.9) G2: 11.2; 3.0 (7.7-16.9)	Medulloblastoma (16) Ependymoma (6) (incl. anaplastic = 4) Germ cell (2) Others (4)	Quasi RCT, crossover, blinded interrater reliability test; Quality 3
Szulc-Lerch et al., 2018, NCT01944761	28	G1: 6.3; 1.6 (2.9-8.1) G2: 5.6; 2.6 (1.9-9.3)	G1: 12; 3.0 (8.1-16.9) G2: 11.2; 3.0 (7.7-16.9)	Medulloblastoma (16) Ependymoma (6) (incl. anaplastic = 4) Germ cell (2) Others (4)	Quasi RCT, crossover; Quality 3
Carlson-Green et al., 2017, none reported	21	6; (1-14)	Time since end of treatment: 5 (1-12)	Medulloblastoma (11) Germ Cell (4) Ependymoma (4) Others (2)	Single arm intervention trial; Quality 2
De Ruiter et al., 2016, NCT00961922	71	G1: 6.8; 3.7 G2: 7.4; 4.1	G1: 14.5; 3.0 G2: 13.5; 3.3	High grade (28) Low grade (43)	RCT, double-blind, parallel placebo controlled; Quality 1
Kasatkin et al., 2022, none reported	63	Not reported	11.6; 3.4	Medulloblastoma (35) Astrocytoma (20) Ependymoma (5) Ganglioglioma (3)	Quasi RCT, crossover; Quality 3
Peterson et al., 2022, NCT02573441	28	G1: 7.9 (2.1-12.1) G2: 4.5 (1.7-11.4) G3: 4.7 (1.1-7.10)	G1: 13.7 (8.8-15.6) G2: 10.6 (7.0-14.7) G3: 10.3 (7.1-14.6)	Medulloblastoma (9) Ependymoma (10) Astrocytic Tumors (3) Others (6)	Pilot RCT, open-label, parallel arm; Quality 3
Siciliano et al., 2022, none reported	41	range (7-16)	G1: 12.0; 2.7 G2: 11.7; 2.8	High grade (19) Low grade (20) Unknown (2)	RCT; Quality 3
Grenawalt et al., 2022, NCT03871686	127	G1: 9.7; 4.3 G2: 9.4; 4.6	G1: 23.8; 3.1, G2: 23.9; 3.0	Ependymoma (32) Embryonal (32) Craniopharyngioma (26) Others (37)	RCT; Quality 3
Poggi et al., 2009, none reported	40	G1: 5.5; 3.8, G2: 7.6; 4.1	G1: 9.6; 4.0 G2: 9.3; 3.2	Medulloblastoma (10) Ependymoma (10) Astrocytoma (8) Others (12)	CT; Quality 3
Sabel et al., 2017, none reported	13	Age at RT G1: 8.0; 3.4 G2: 8.9; 1.6	G1: 11.9; 3.6 G2: 13.2; 1.9	Medulloblastoma (3) Germ cell (3) Pineoblastoma (3) Others (4)	RCT, crossover; Quality 3
Wade et al., 2019, none reported	19	Time since diagnosis: 8.9; 4	17.6; 4	Astrocytoma/glioma (11) Ependymoma (5) Others (3)	Single arm intervention trial; Quality 2

CT, controlled trial; G1-3, group 1-3; N, number; NCT, unique clinical trial number on ClinicalTrials.gov; RCT, randomized controlled trial; RT, radiotherapy; SD, standard deviation.

The 14 included publications studied different types of interventions to prevent or improve neurocognitive late-effects in children or adolescents diagnosed with CNS tumors. The reported interventions can be divided into pharmacological interventions (11, 23), exercise interventions (16, 18, 19), online cognitive training (12, 15, 17, 20, 22), and behavioral interventions (13, 14, 21, 24). The intervention and outcomes are summarized in **Table 2**.

**Table 2:** Interventions to improve neurocognitive outcomes and impact of intervention.

Author, Year of publication	Intervention group	Control group	Timepoint of assessment - Assessment of neurocognitive outcomes		Impact of intervention on neurocognitive outcomes
Pharmacological interventions					
Ayoub et al., 2020	12 weeks Metformin (500-1000mg/m^2^ once daily)	Placebo plus crossover	Assessment at week 12 - Working memory: LSWM - Visual memory: PSM from the NIH Toolbox - Declarative memory: CAVLT-2, RAVLT - Information processing speed: average reaction time across select tests from the CANTAB		- Working memory: increased correct answers in LSWM and decreased average latency on CANTAB in intervention group in univariable but not multivariable analysis - All effects showed trend for positive impact of metformin
Castellino et al., 2012	24 weeks Donepezil (≥ 35 kg: 5-10mg once daily; < 35 kg: 5mg every 2 days to once daily)	No control group	Assessment at week 24 - Executive function: D-KEFS - Memory: WRAML 2nd Edition - Attention: CPT II - Processing speed: WISC-IV symbols search subtest - Achievement: Woodcock Reading Mastery Test; WJ-III Calculations - Parents reported measures: executive function (BRIEF), behavior (BASC-2), and family function - Child and parent proxy report on HRQL		- Executive functioning: Increased performance accuracy (D-KEFS tower task), increased time-per-move ratio (D-KEFS towers time ratio), increased verbal inhibition, and simultaneous processing and cognitive flexibility (D-KEFS color/word interference inhibition) - No changes in letter fluency and sorting tasks - Increased visual memory composite score and number/letter memory (WRAML-2) - No significant impact on sustained attention and concentration, processing speed, and achievement - Parent report: increased plan/organizing skills, working memory, and emotional control
Exercise interventions					
Cox et al., 2020	90 min group-based aerobic activities Group: 3x/week Group/home: 2x/week plus two individual home sessions both for 12 weeks	No training	Assessment at week 12 and 24 - Attentional control - Completion of Go and Go/No-Go tasks during magnetoencephalography		- Improvement in response accuracy during Go/No-Go trials; no impact on response latency
Riggs et al., 2017	90 min group-based aerobic activities Group: 3x/week Group/home: 2x/week plus two individual home sessions both for 12 weeks	No training	Assessment at week 12 and 24 - White matter architecture (FA) and hippocampal volume in T1 MRI - CANTAB: attention (rapid visual information processing, match to sample visual search), processing speed (simple reaction time, choice reaction time), and short-term memory (delayed matching to sample)		- Increased FA across the corpus callosum, cingulum, superior longitudinal fasciculi bilaterally, right corticospinal tract, and inferior frontal occipital fasciculus through training in the group setting - Increased total hippocampal volume in the group setting - Decreased reaction time in group setting
Szulc-Lerch et al., 2018	90 min group-based aerobic activities Group: 3x/week Group/home: 2x/week plus two individual home sessions both for 12 weeks	No training	Assessment at week 12 and 24 - Changes in cortical thickness and volume in T1 MRI - CANTAB: attention (rapid visual information processing, match to sample visual search), processing speed (simple reaction time, choice reaction time), and short-term memory (delayed matching to sample, verbal recognition memory) - Motor function: BOT-2 - 6MWT - CDI-2		- Increased cortical thickness for right precentral and postcentral gyri in group setting (only precentral gyrus after Bonferroni correction) - Increased cortical thickness for bilateral postcentral gyrus in combined setting Increased white matter volume: right motor and somatosensory cortex, right parietal lobe - Increased right hemispheric cortical thickness associated with improved performance on metric measures and changes in accuracy and reaction time - Increased cortical thickness across entire cortex associated with decreased scores on the CDI–2, decreased reaction, improved scores on short-term memory tasks, and improved physical functioning
Online cognitive training					
Carlson-Green et al., 2017	Cogmed® Working MemoryTraining, extended 35 sessions for 8 to 12 weeks	No control group	Assessment at baseline, completion of training and 6-months - Working memory: AWMA - Academic achievement: WJ-III - Parents completed questionnaires: behavior problems, emotional problems (CBCL), executive functioning (BRIEF), adaptive functioning and social skills (ABAS-II), exposure to neurocognitive risk factors (Neurological Predictor Scale)	- Improved scores on 2 of 3 verbal working memory tasks - Improvements on 4 of 5 visual-spatial working memory tasks - Improvement in WJ-III in math - Parents reported improved executive functioning subscales on working memory (BRIEF: inhibitory control, self-monitoring, planning/organization), reduced somatic symptoms and attention problems (CBCL), and improved social skills (ABAS-II)	
De Ruiter et al., 2016	Neurofeedback: 30 sessions of 30 minimum twice weekly for 3 months	Placebo feedback over 15 weeks	Assessment pre training (T0), directly post training (T1), and six months post training (T2) - Attention: ANT - Processing speed: mean reaction time on correct answers on ANT trials - Visual short-term memory: visual sequencing task - Auditory short-term memory: WISC-III (forward items digit span task) - Working memory: WISC-III (backward items digit span task) - Response inhibition: stop signal task - Visuomotor integration: tracking and pursuit task - Intelligence: WISC-III, WAIS	- No effect on attention, processing speed, memory, executive functioning, visuomotor integration, and intelligence	
Kasatkin et al., 2022	Cognitive and motor training by Dynavision D2®, Fitlight Trainer® or NeuroTrackerX®, 6 to 8 sessions over 2 weeks	No intervention plus crossover	Assessment at week 2 and week 4 - Cognitive functions by CANTAB: spatial working memory, spatial recognition memory, pattern recognition memory, spatial span, rapid visual information processing, stocking of Cambridge - Motor function: BOT-2 - Visual-motor integration: Beery VMI - Saccadic eye movement by Arington eye tracker	- Improvement in gross and fine motor skills, motor coordination, visual-motor integration and visual processing	
Peterson et al., 2022	Cogmed® Working MemoryTraining or JumpMath® 12-weeks home-based intervention with weekly telephone-based psychological consultation	Active control = reading and weekly telephone-based psychological consultation	Assessment at baseline and week 12 - Working memory: WISC-IV, WRAML-2 - Academic achievement and mathematic skills: WJ-III	- Increased backward digit span and symbolic working memory but not letter number sequencing and verbal working memory in Cogmed® and JumpMath® - JumpMath® group improved in calculation subtest compared to control. No difference for Cogmed	
Siciliano et al., 2022	Cogmed® Working MemoryTraining, adaptive version (adjusted to daily performance) 30-45 minimum 5 days per weeks over 5 weeks (total of 25 sessions)	Active control = Cogmed® nonadaptive version	Assessment at baseline (T1), 5-8 weeks postintervention (T2), 10–20 weeks postintervention (T3), 6 months after the previous assessment (T4) - General Intellectual functioning: WASI-II, WISC-IV, WMI - 5 subtests of NTCB: - Cognitive flexibility and attention: dimensional change card sort test - Executive function and inhibitory control: Flanker inhibitory control and attention test - Working memory: LSWM - Processing speed: pattern comparison processing speed test - Episodic memory: picture sequence memory test - Parent reported function and attention: BRIEF, CBCL	- No evidence of greater improvement over time in adaptive vs. nonadaptive Cogmed® - Improvement in WMI and the NTCB from baseline (T1) to immediately postintervention (T2), but no change in WMI at both follow-up points at 10 to 20 weeks (T3) and 6 months (T4) compared to T1	
Behavioral interventions					
Grenawalt et al., 2022	Internet-based behavioral activation intervention (incl. values assessment, mindfulness exercises, social skills education)	Services as usual	Assessment pre and post test - QOL and life satisfaction (Life Satisfaction Questionnaire (LiSat-9)) - Stress (Perceived Stress Scale (PSS-10)) - Activation (Behavioral Activation for Depression Scale – Short form (BADS-SF))	- Study participants in the experimental group demonstrated a significant gain in life satisfaction compared to the control group - Perceived stress declined in both groups - Both groups demonstrated declines in activation, but only the waitlist control group had a significant decline	
Poggi et al., 2009	Cognitive behavioral therapy, 2 to 3 sessions for 4 to 8 months	No therapy	Assessment at 12 months - CBCL - Vineland Adaptive Behavioral Scales (VABS)	- CBCL: significant decrease in the intervention versus control group for withdrawn, somatic complaints, social problems, attention problems, internalizing, overall less problems - CBCL: no significant change for anxiety/depression, thought problems, delinquent behavior, aggressive behavior, externalizing - VABS: better social skills in intervention group, no change for communication, social skills, motor skills	
Sabel et al., 2017	Active video gaming (motion-controlled video console Nintendo^®^ Wii™), minimum. 30 min a day, 5 days a week and weekly internet-based coaching sessions for 10 to 12 weeks	No gaming and crossover	Assessment at study start, in-between periods, and at the end of study - Execution of activities of daily living (assessment of Motor and Process Skills) - Cognitive tests: - Attention: CPT II, Map mission, visual scanning from D-KEFS - Verbal working memory: digit span (WISC-IV), auditory consonant trigrams, RAVLT - Visuospatial working memory: spatial span (WNV) - Verbal learning and verbal long-term memory: RAVLT, WISC-IV - Visuospatial long-term memory: Rey complex figure test - Information processing speed: WISC-IV - Executive function: controlled oral word association test, Stroop test, trail making test, switching condition (D-KEFS) - Social Competence: picture arrangement (WNV) - General ability: WISC- IV (4 subtests)	- Motor and process parts improved after video gaming - No change in cognitive tests - Trends for improvement in sustained attention (CPT-II omission) and selective attention (Map mission)	
Wade et al., 2020	A Survivors Journey online intervention. online module, weekly trained therapist meetings on Skype for 2 to 4 months	No control group	Assessment at baseline and 2 – 4 months postbaseline - Depression: CES-D - Executive function: BRIEF - Quality of life: PedsQL - Intellectual functioning: WASI	- Depression: no effect - Executive function: no effect - Quality of Life: improvement (overall, physical, emotional, social all improved), - Patients with higher IQ in the beginning reported improvement from pre- to post-intervention	

ABAS-II, Adaptive Behavior Assessment System, 2nd Ed.; ANT, Attention Network Task; AWMA, Automated Working Memory Assessment, BASC-2, Behavior Assessment System for Children, Second Edition; BOT, Bruininks-Oseretsky Test of Motor Proficiency; BRIEF, Behavior Rating Inventory of Executive Function; CANTAB, Cambridge Neuropsychological Test Automated Battery; CAVLT-2, Children’s Auditory Verbal Learning Test-2; CBCL, Child Behavior Checklist; CDI-2, Children’s Depression Inventory (2nd Edition); CES-D, Center for Epidemiologic Studies Depression Scale; CPT, Conners Continuous Performance Test; D-KEFS, Dellis-Kaplan Executive Function Test; FA, fractional anisotropy, LSWM List Sorting Working Memory; NIH, National Institutes of Health; NTCB, National Institute of Health Toolbox Cognitive Battery; min, minutes; 6MWT, 6–Minute Walk Test; MRI, Magnetic resonance imaging; PSM, Picture Sequence Memory Test; QOL, Quality of life; RAVLT, Rey Auditory Verbal Learning Test; VMI, Visual Motor Integration; WAIS, Wechsler Adult Intelligence Scale; WISC, WNV, Wechsler nonverbal scale of abilities; WMI, Working Memory Index, Wechsler Intelligence Scale for Children; WJ-III, Woodcock Johnson III; WRAML, Wide Range Assessment of Memory and Learning.

### 3.1 Pharmacological interventions

Two studies investigated pharmacological interventions. Ayoub et al. studied metformin and found a trend for increased working memory and information processing speed if administered over 12 weeks (11). According to Castellino et al, donepezil increased executive functioning and visual memory as well as number/letter memory in a single-arm intervention trial (23).

### 3.2 Exercise interventions

Three publications reported different outcomes of the same quasi-randomized controlled trial with crossover design on aerobic exercise training (study identifier: NCT01944761) (16, 18, 19). The group studied the impact of repeated group-based or mixed group- and home-based aerobic activities three times a week for 12 weeks. One publication showed improved response accuracy in those with training compared to those without (16). The second publication reported increased white matter architecture and hippocampal volume in those with training (18). When comparing the group-based and the mixed group- and home-based setting, the group-based setting decreased reaction time as an indicator for improved processing speed. The third publication found increased cortical thickness across the entire cortex on imaging (19). This was associated with improved scores on short-term memory tasks and improved physical functioning as well as a decrease in depression and reaction time. The increase in right hemispheric cortical thickness on the other hand was associated with an improvement of metric measures and accuracy in reaction time (19).

### 3.3 Online cognitive training

Five publications reported on online cognitive training (12, 15, 17, 20, 22). Most of them reported on commercially available online programs. Carlson-Green et al. found some improvement in verbal and visual working memory tasks as well as improvement in academic achievement after 35 sessions of Cogmed® Working Memory Training over 8-12 weeks (22). In the study by Siciliano et al., Cogmed® improved working memory right after the end of the intervention but not in the longer term. It made no difference whether the adaptive or non-adaptive version of the program was used (15). Cogmed® performed similarly to JumpMath® with an increase of working memory in the study by Peterson et al. (20). Additionally, calculation subtests improved in persons using JumpMath® compared to those working with Cogmed® (20). De Ruiter et al. compared online neurofeedback to placebo feedback in 30 sessions over three months and found no effect on attention, processing speed, memory, executive functioning, visuomotor integration, and intelligence (12). Kasatkin et al. showed in a crossover trial that cognitive and motor training with Dynavision D2®, Fitlight Trainer® and NeuroTrackerX® in 6 to 8 sessions over 2 weeks improved gross and fine motor skills, motor coordination, visual-motor integration, and visual processing in the intervention group.

### 3.4 Behavioral interventions

Four publications reported on behavioral interventions, three were internet-based programs (13, 14, 24) and one was a person-to-person cognitive behavioral therapy program (21). According to Grenawalt et al. the behavioral activation intervention increased life satisfaction and slowed down activation in the intervention group (13). When combining active gaming and internet-based coaching sessions, Sabel et al. could show improvement in motor and processing skills but not in cognitive tests (14). Wade et al. used “A Survivors Journey” online modules and could show improved quality of life in participants but no improvement in executive or intellectual functioning (24). The more traditional cognitive behavioral therapy sessions studied by Poggi et al. reduced attention problems in the intervention group (21).

## 4 Discussion

We found 14 publications reporting on interventions to mitigate or improve neurocognitive late-effects in pediatric or adolescent CNS tumor patients and survivors and could identify four different types of interventions: pharmacological interventions, exercise interventions, online cognitive training, and behavioral interventions. The effects of most interventions were rather small and inconsistent in comparison. In addition, the approaches used to measure the outcomes differed and direct comparison of different interventions was not possible.

### 4.1 Pharmacological interventions

The studies on pharmacological interventions examined well known drugs with good safety profiles. However, both studies were small with less than 25 participants each, and not optimally designed. Ayoub et al. studied metformin, a drug known as first-line treatment in diabetes mellitus type 2 (25) and experimentally used in the treatment of Alzheimer’s disease associated with diabetes mellitus type 2 (26). The randomized-controlled pilot-feasibility study of Ayoub tried to overcome the small participant number by setting up a crossover design. Nevertheless, no robust effects of metformin on neurocognitive late-effects in pediatric CNS tumor survivors could be observed in the multivariable analysis, only a trend (11). An explanation for the lack of a clear effect of metformin in CNS tumor survivors could be that these patients do not suffer from diabetes mellitus type 2 and that the improvement in patients with Alzheimer’s disease is a secondary effect of controlled metabolism and not due to the direct effects of metformin on the brain itself. Castellino et al. studied donepezil, a cholinesterase inhibitor used in the treatment of dementia where it showed some improvement in executive functioning, cognitive abilities, and behavioral symptoms (27). Even though the investigators found an increase in executive functioning and memory in CNS tumor survivors, the single-arm intervention trial cannot distinguish the drug effects on neurocognitive problems from improvement through other parallel supports that have not been studied (e.g., support at school, rehabilitation programs) (23). Both interventions need to be reevaluated in larger populations with comparison groups.

### 4.2 Exercise interventions

We could identify one trial reporting different aspects of neurocognition including anatomical changes in the brain after exercise intervention in three separate publications (16, 18, 19). The trial was relatively small with only 28 participants. The hypothesis of the trial was that higher fitness levels might improve cognitive function in pediatric CNS tumor survivors due to improved organization of white matter and increased cortical volumes. This effect was shown in healthy children before (28). Similar effects could also be shown in the three publications indicating that physical exercise should be promoted in survivors of pediatric and adolescent CNS tumors. This trial only examined aerobic exercise activities, but non-aerobic either high-intensity or low-intensity exercise were not studied. It might be helpful to evaluate these modalities in the future because not all pediatric CNS tumor survivors will be able to participate in aerobic exercise training.

### 4.3 Online cognitive training

Cogmed® Working MemoryTraining was the most frequently studied application for online cognitive training (29). Cogmed® is a computer game-like training program claiming to improve working memory and attention with a non-adaptive and an adaptive version available. The adaptive version adjusts the tasks to the participants daily performance. The included studies showed that working memory improved but improvements did not differ much when compared to other home-based online cognitive trainings such as JumpMath® (20, 30). Additionally, the adaptive version of Cogmed® did not influence the improvement compared to the non-adaptive version (15). Other cognitive training such as the combination of Dynavision D2® (31), Fitlight Trainer® (32), and NeuroTrackerX® (33) also showed cognitive improvement. These results suggest that online cognitive programs improve the working memory, but that the program itself does not matter, as long as there is some online cognitive training done. If online cognitive training is used, we suggest keeping to local practices and availabilities.

### 4.4 Behavioral interventions

Few to no effects on neurocognitive problems in pediatric CNS tumor survivors could be observed in all three internet-based behavioral interventions (13, 14, 24). On the other hand, Poggi et al. could show that traditional person-to-person cognitive behavioral therapy decreased attention problems. These findings differ from the general population where person-to-person and internet-based behavioral interventions were similarly effective (34). One possible explanation being that children and adolescents might already have had contact with psychological liaison service during diagnosis and treatment and are already used to person-to-person contact. Additionally, fatigue is a common late-effect after treatment of CNS tumors and might influence concentration and perseverance in internet-based interventions (35).

### 4.5 Implications for research and practice

In this review we focused on interventions aiming to improve neurocognitive problems in pediatric and adolescent CNS tumor patients or survivors. The 14 included studies used many different neurocognitive assessment tools leading to a variability in the assessment of success of the studied interventions. The variety in interventions and outcome measures made direct comparison difficult and prohibits an estimate of superiority or inferiority of the investigated interventions. A further limitation of the included publications were the small sample sizes. Consequently, the findings often resulted only in the reporting of trends or small effects of the studied interventions. For future research we recommend studying larger patient numbers and to perform preferably international and harmonized collaborations to assess efficacy of interventions to mitigate neurocognitive problems in pediatric and adolescent CNS tumor patients or survivors.

## 5 Conclusion

We found several publications indicating improvement of neurocognitive problems in pediatric and adolescent CNS tumor survivors like exercise interventions or online cognitive training. Both intervention types can be implemented relatively easily in daily practice if respective resources are available or via remote access from home. Aerobic exercise training can be instructed by local physiotherapists in group or personalized settings. For online cognitive training interventions we suggest keeping to local practices and availabilities as there seems to be no big difference in the outcomes between the different programs examined.

## Author contributions

RK: acquisition, analysis, interpretation of data, writing manuscript and final approval of manuscript. PF: acquisition, analysis, interpretation of data, writing manuscript and final approval of manuscript. MO: acquisition, analysis, interpretation of data, review of manuscript and final approval of manuscript. KS: conception of work, acquisition, analysis, interpretation of data, review of manuscript and final approval of manuscript. All authors contributed to the article and approved the submitted version.
