# Supplementary material for: Interventions to improve neurocognitive late-effects in pediatric and adolescent CNS tumor patients and survivors - a systematic review
Source: Front Oncol. 2023 May 2;13:1150166. doi: 10.3389/fonc.2023.1150166 (PMC10185878; doi:10.3389/fonc.2023.1150166)
Supplement: Supplementary file 1 [file DataSheet_1.pdf]

Supplementary Material

Interventions to improve neurocognitive late-effects in pediatric and adolescent CNS tumor patients and survivors - a systematic review

Rahel Kasteler[1*], Philipp Fuchs[1], Maria Otth[2, 3±], Katrin Scheinemann [3,4,5±]

*** Correspondence:** Rahel Kasteler: rahel.kasteler@ksa.ch

[1] Division of Pediatric Hematology/Oncology, Department of Pediatrics, Kantonsspital Aarau, Aarau, Switzerland.

[2] Department of Oncology, Hematology, Immunology, Stem Cell Transplantation and Somatic Gene Therapy, University Children's Hospital Zurich, Zurich, Switzerland.

[3] Division of Pediatric Hematology/Oncology, Children's Hospital of Eastern Switzerland, St. Gallen, Switzerland.

[4] Department of Health Sciences and Medicine, University of Lucerne, Lucerne, Switzerland.

[5] Division of Pediatric Hematology/Oncology, McMaster Children's Hospital and McMaster University, Hamilton, ON, Canada.

[±] Shared last author

Supplementary Table 1 PICO terms for systematic review and PubMed Search strategy

**PICO terms**	
Population 1	central nervous system tumors (including specific types of CNS tumours but also broad terms)
Population 2	children and adolescents
Population 3	survivors
Population 4	patients
Intervention	-
Control	-
Outcome 1	neurocognition
Outcome 2	interventions

**PubMed Search Strategy**	
16	#11 AND #12 AND #15 AND #9 AND #10
15	#13 OR#14
14	#7 OR #8
13	#5 OR #6
12	#3 OR #4
11	#1 OR #2
10	intervent*[tiab] OR prevent*[tiab] OR improv*[tiab]
9	Neurocogn*[tiab] OR cogniti*[tiab]
8	patient*[tiab]
7	Patient[MesH]
6	Surviv*[tiab] OR remission*[tiab] OR aftercare[tiab] OR (After Care)[tiab] OR (follow up)[tiab] OR followup[tiab] OR longterm[tiab] OR long-term[tiab] OR (late effect)*[tiab] OR late-effect*[tiab] OR sequela*[tiab]
5	Survivors[Mesh] OR Survival[Mesh] OR Long-Term Care[Mesh] OR Aftercare[Mesh] OR Disease-Free Survival[Mesh]
4	child*[tiab] OR infan*[tiab] OR adolescen*[tiab] OR newborn*[tiab] OR neonat*[tiab] OR schoolchild*[tiab] OR school child*[tiab] OR toddler*[tiab] OR teen*[tiab] OR youth*[tiab] OR young*[tiab] OR kindergar*[tiab] OR pediatric*[tiab] OR paediatric*[tiab] OR school*[tiab] OR preschool*[tiab] OR pre-school*[tiab] OR elementary school*[tiab] OR highschool*[tiab] OR high-school*[tiab] OR schoolage*[tiab] OR (school age)[tiab]
3	Child[MesH] OR Infant[MesH] OR Adolescent[MesH] OR Pediatrics[MesH]
2	astrocytom*[tiab] OR craniopharyngeom*[tiab] OR medulloblastom*[tiab] OR PNET*[tiab] OR (neuroectodermal [tiab] AND tumor*[tiab]) OR gliom*[tiab] OR (brain [tiab] AND tumor*[tiab]) OR (brain [tiab] AND tumour*[tiab]) OR (intracranial [tiab] AND tumor*[tiab]) OR (intracranial[tiab] AND tumour*[tiab]) OR ((central [tiab] AND nervous [tiab] AND syst*[tiab]) AND tumor*[tiab]) OR ((central [tiab] AND nervous [tiab] AND syst*[tiab]) AND tumour*) OR (CNS [tiab] AND tumor*[tiab]) OR (CNS [tiab] AND tumour*[tiab]) OR(brain [tiab] AND cancer*[tiab]) OR (intracranial [tiab] AND cancer*[tiab]) OR ((central [tiab] AND nervous [tiab] AND syst*[tiab]) AND cancer*[tiab]) OR (CNS [tiab] AND cancer*[tiab]) OR (brain [tiab] AND malignanc*[tiab]) OR (intracranial [tiab] AND malignanc*[tiab]) OR ((central [tiab] AND nervous[tiab] AND syst*[tiab]) AND malignanc*[tiab]) OR (CNS [tiab] AND malignanc*[tiab]) OR (brain [tiab] AND neoplas*[tiab]) OR (intracranial [tiab] AND neoplas*[tiab]) OR ((central [tiab] AND nervous [tiab] AND syst*[tiab]) AND neoplas*[tiab]) OR (CNS [tiab] AND neoplas*[tiab])
1	Central Nervous System Neoplasms [MesH]

Supplementary Table 2 Critical appraisal of studies included in final systematic review according to Joanne Briggs Institute

Randomized Controlled Trials

Author	Year	Was true randomization used for assignment of participants to treatment groups?	Was allocation to treatment groups concealed?	Were treatment groups similar at the baseline?	Were participants blind to treatment assignment?	Were those delivering treatment blind to treatment assignment?	Were outcomes assessors blind to treatment assignment?	Were treatment groups treated identically other than the intervention of interest?	Was follow up complete and if not, were differences between groups in terms of their follow up adequately described and analyzed?	Were participants analyzed in the groups to which they were randomized?	Were outcomes measured in the same way for treatment groups?	Were outcomes measured in a reliable way?	Was appropriate statistical analysis used?	Was the trial design appropriate, and any deviations from the standard RCT design (individual randomization, parallel groups) accounted for in the conduct and analysis of the trial?	Quality over All
Ayoub	2020	quasi randomly [a]	no	no but crossover	yes	yes	yes	yes	yes	yes	yes	yes	yes	yes	Quality 2
Cox	2020	quasi-randomly [a]	no	no but crossover	no	no	no	yes	no	no [b]	yes	yes	yes	yes	Quality 3
de Ruiter	2016	yes	yes	yes	yes	yes	yes	yes	yes	yes	yes	yes	yes	yes	Quality 1
Grenawalt	2022	yes	yes	yes	no	no	unclear	no	no	yes	yes	yes	yes	yes	Quality 3
Kasatkin	2022	quasi-randomly	no	yes, crossover	no	no	unclear	yes	not explained	yes	yes	yes	yes	no wash-out	Quality 3
Peterson	2022	unclear	yes	no	no	no	yes	yes	yes	yes	yes	yes	yes	yes	Quality 3
Poggi	2009	no	no	no	no	no	unclear	no	yes	yes	yes	yes	yes	yes	Quality 3
Riggs	2017	quasi-randomly	no	no but crossover	no	no	yes	yes	yes	no [b]	yes	yes	yes	yes	Quality 3
Sabel	2017	yes	yes	no but crossover	no	no	yes	yes	yes	no [b]	yes	yes	yes	yes	Quality 3
Siciliano	2022	block randomized	yes	no	yes	unclear	unclear	yes	no	yes	yes	yes	yes	yes	Quality 3
Szulc-Lerch	2018	quasi-randomly	no	no but crossover	no	no	unclear	yes	no	no [b]	yes	yes	yes	yes	Quality 3

[a] based on the order they were recruited
[b] only training vs no training

Quasi-Experimental

Author	Year	Is it clear in the study what is the `cause’ d what is the `effect’ (i.e. there is no confusion about which variable comes first)?	Were the participants included in any comparisons similar?	Were the participants included in any comparisons receiving similar treatment/care, other than the exposure or intervention of interest?	Was there a control group?	Were there multiple measurements of the outcome both pre and post the intervention/exposure?	Was follow up complete and if not, were differences between groups in terms of their follow up adequately described and analyzed?	Were the outcomes of participants included in any comparisons measured in the same way?	Were outcomes measured in a reliable way?	Was appropriate statistical analysis used?	Quality over All
Carson-Green	2017	yes	yes	yes	no	yes	yes	yes	yes	yes	Quality 2
Castellino	2012	yes	yes	yes	no	yes	yes	yes	yes	yes	Quality 2
Wade	2020	yes	yes	yes	no	yes	no	yes	yes	yes	Quality 2
